# From spatial intervention to healthy ageing: proposing a Spatial–Social–Ecological framework for active ageing community renewal

**DOI:** 10.3389/fpubh.2026.1909608

**Published:** 2026-09-01

**Authors:** Min Zhou, Yang Li, Jing Huang, Simeng Liu

**Affiliations:** College of Architecture and Art Design, Henan Polytechnic University, Jiaozuo, Henan, China

**Keywords:** active-ageing community renewal, age-friendly communities, bibliometric analysis, built environment, community health, healthy ageing, social capital, therapeutic landscape

## Abstract

**Introduction:**

Community renewal oriented toward active ageing has become an important strategy for addressing population ageing and urban regeneration. However, existing research often examines spatial, social and ecological factors separately, resulting in a limited understanding of how community renewal can promote sustainable health outcomes.

**Method:**

This bibliometric analysis followed PRISMA 2020-guided procedures for literature identification and screening and analyzed 1,386 publications (716 international and 670 Chinese) published between 2015 and 2025, retrieved from the Web of Science Core Collection (SSCI, SCIE, A&HCI) and the China National Knowledge Infrastructure (CNKI).

**Results:**

(1) The focus of research has clearly shifted from facility-oriented interventions to integrated approaches that encompass spatial, social and ecological aspects. (2) A conceptual Spatial–Social–Ecological (SSE) framework is proposed to organize the hypothesized relationships among environmental interventions, social participation, ecological healing, and healthy ageing.

**Discussion:**

The proposed framework provides an evidence-informed theoretical perspective for understanding how community renewal may support healthy ageing and offers a conceptual basis for future empirical research and evidence-based community renewal policies.

## 1 Introduction

The World Health Organization (WHO) has introduced the concept of “active ageing,” emphasising the need to optimise opportunities for health, participation and safety to enhance quality of life across the life course (1). As communities constitute the primary spatial setting in which ageing in place occurs, community renewal has increasingly been recognised as one of the most practical policy approaches for translating healthy ageing principles into neighbourhood-level environmental interventions. As the primary setting for ageing in place, communities play a crucial role in supporting healthy ageing by shaping older adults’ daily activities, social interactions, and environmental experiences (2). Consequently, community renewal guided by the principles of active ageing has become a key strategy for addressing the dual challenges of population ageing and urban community renewal.

Existing research has extensively analysed the relationship between the community environment and healthy ageing. From the perspective of the built environment, studies have examined how accessibility, walkability, housing conditions, and public facilities influence older adults’ physical activity, mobility, and health outcomes (3–5). From the perspective of social governance, social participation, neighbourhood support, place attachment, and community governance are receiving increasing attention as mechanisms that promote active ageing (6–8). In recent years, the roles of the natural environment, green infrastructure, and environmental quality in promoting mental health and healthy ageing have gained increasing recognition (9–11). Despite these advances, the existing literature often examines spatial, social and ecological dimensions in isolation, rather than exploring them as interrelated factors influencing long-term health outcomes. At the international level, the World Health Organization (WHO) World Report on Ageing and Health redefined healthy ageing as the result of the interaction between an individual’s functional capacity and environmental conditions, thereby establishing environmental interventions as a core component of ageing policies (1). In China, the Central Urban Work Conference accelerated the transition from large-scale urban expansion to stock-oriented urban renewal, positioning community renewal as a key factor in improving public services and social governance (12). The convergence of global and national trends has created a critical opportunity to integrate research on ageing policies, public health, and community renewal (13). Consistent with this policy context, the bibliometric evidence synthesized in this review also indicates a rapid increase in studies concerning community renewal, ageing communities, and neighbourhood governance, highlighting the growing need for an evidence-informed theoretical framework for high-density older residential communities. In addition to compact spatial morphology, Chinese high-density older residential communities are characterized by complex governance arrangements involving local governments, neighbourhood committees, property management organizations, and residents. These communities also face ageing-related challenges, including declining accessibility, insufficient spaces for social interaction, and limited restorative environmental resources. Therefore, their renewal requires an integrated perspective that considers spatial, social, and ecological dimensions simultaneously.

However, three main challenges can be identified. First, spatial, social and ecological variables are primarily viewed as parallel determinants rather than theoretical relationships, leading to an incomplete understanding of how community renewal interventions translate into sustainable health outcomes. Second, the therapeutic dimension of the community environment has received limited attention in community renewal research, particularly in high-density urban settings. Although “Therapeutic Landscape Theory” (13), “Attention Restoration Theory” (14), and “Stress Reduction Theory” (15) offer valuable explanations for environmental restoration and healthy ageing, these perspectives have rarely been incorporated into community renewal research oriented toward active ageing. Finally, a unified framework linking spatial adaptation, social embeddedness, and ecological healing has yet to emerge.

From a public health perspective, ageing-friendly community renewal should not be understood solely as a physical improvement strategy. Increasing evidence suggests that neighbourhood environments influence older adults’ health through multiple pathways, including physical activity promotion, mobility maintenance, psychological restoration, social interaction, and subjective wellbeing. Environmental barriers, inadequate accessibility, limited opportunities for social participation, and insufficient restorative environments may increase health risks among older adults, including reduced mobility, increased loneliness, psychological stress, and decreased quality of life.

To address these gaps, this study conducted a bibliometric analysis of active ageing-oriented community renewal research published between 2015 and 2025, retrieving literature from the Web of Science Core Collection and China National Knowledge Infrastructure (CNKI). Through CiteSpace-based analysis, major research hotspots, thematic evolution, and research frontiers were identified. Based on the bibliometric findings, this study synthesises Person--Environment Fit Theory, Social Capital Theory and Therapeutic Landscape Theory into an evidence-informed conceptual Spatial–Social–Ecological (SSE) framework to organise the hypothesised relationships among environmental interventions, social participation, ecological healing and healthy ageing. Rather than representing an empirically validated causal model, the proposed framework provides a conceptual basis for future empirical research and evidence-based community renewal practice.

This study addresses the following research questions: *RQ1*: Through bibliometric analysis, what are the main evolutionary trajectories and thematic clusters in research on community renewal oriented toward active ageing between 2015 and 2025? *RQ2*: How can spatial adaptation, social embeddedness, and ecological healing be theoretically integrated into an interdisciplinary explanatory framework to account for the effects of community health? *RQ3*: What are the boundary conditions and operationalization requirements for applying the Spatial–Social–Ecological (SSE) framework to high-density older residential communities in China?

## 2 Materials and methods

**Figure 1:**
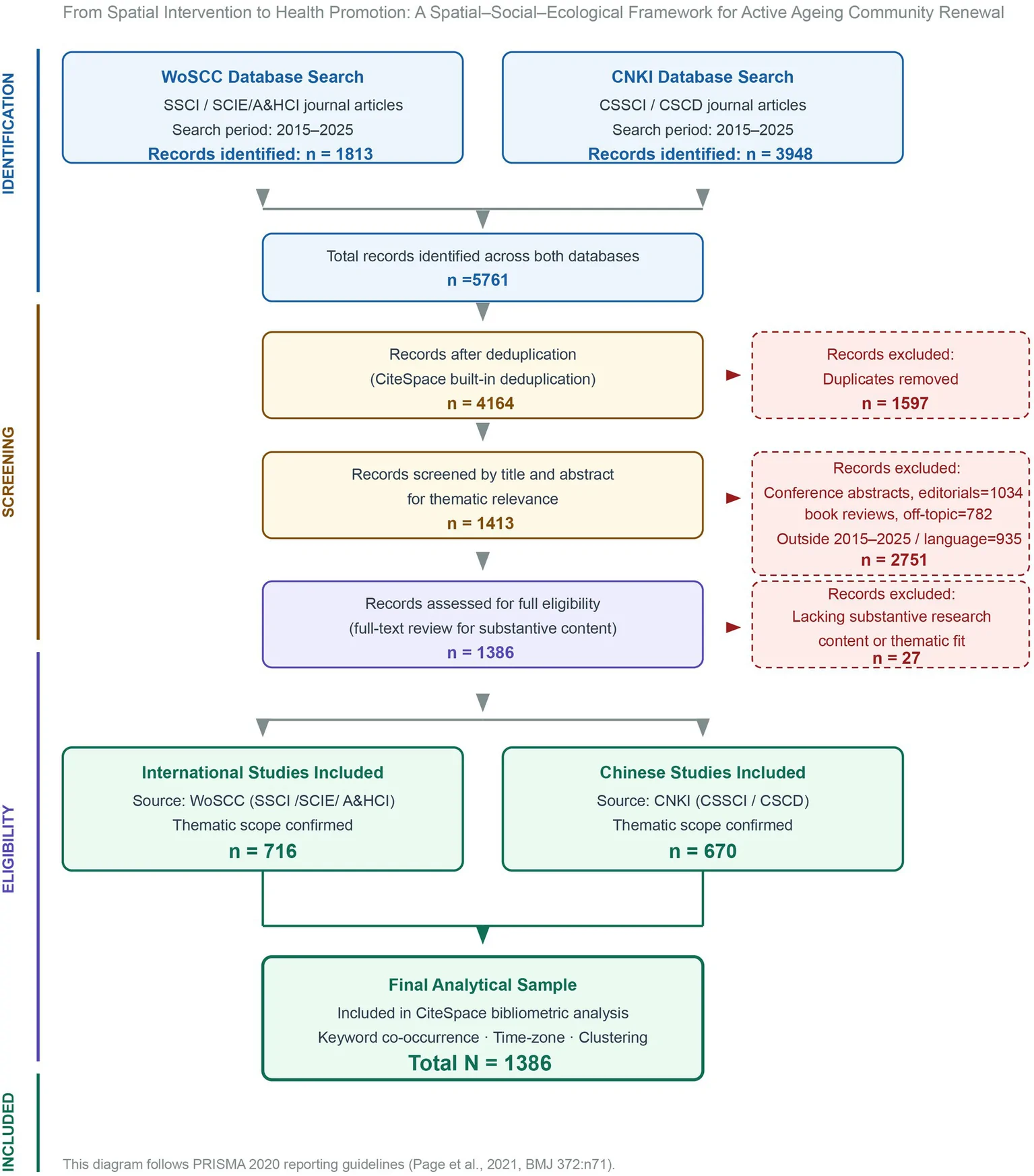
PRISMA flowchart.

This study is designed as a bibliometric analysis employing PRISMA 2020-guided literature identification and screening procedures (Figure 1). PRISMA 2020 was adopted to ensure a transparent and reproducible process for literature identification and screening.

### 2.1 Database selection and scope of the study

To comprehensively analyze research on urban community renewal guided by active ageing, the databases selected were the Web of Science Core Collection (WoSCC) and China National Knowledge Infrastructure (CNKI). Using literature retrieved from these two databases, this study comparatively examined the development of international and Chinese research on active ageing, community renewal, and the built environment. WoSCC was selected because it includes high-quality journals indexed in SSCI, SCIE and A&HCI and is widely recognised as an authoritative source for identifying international research trends and theoretical developments. CNKI was included because it comprehensively covers Chinese academic publications and contains policy-oriented research related to urban renewal, community governance, and age-friendly renovation practices.

The study focuses on literature published between 2015 and 2025. The year 2015 was chosen as the starting point because it marked a significant turning point at both the international and Chinese policy levels. Internationally, the World Health Organization’s World Report on Ageing and Health emphasized the role of environmental conditions in supporting healthy ageing (1). In China, the Central Urban Work Conference signalled a shift in urban development from expansion-oriented growth to urban regeneration and community renewal (12). These developments collectively provide the policy and research context for the integration of research on ageing, health, and community renewal.

### 2.2 Literature search and screening process

We conducted a literature search in the WoSCC and CNKI databases using a predefined search strategy. For WoSCC, the search criteria were set as follows: TS = (“active ageing” OR “active aging” OR “age-friendly”) AND TS = (“community renewal” OR “urban renewal” OR “community governance” OR “built environment”). The search scope was limited to journal articles indexed in SSCI, SCIE and A&HCI. For CNKI, the following terms were used in the title, keyword and abstract fields: (“积极老龄化” OR “老年友好”) AND (“社区更新” OR “城市更新” OR “社区治理” OR “建成环境”) [English gloss: (“active ageing” OR “age-friendly”) AND (“community renewal” OR “urban renewal” OR “community governance” OR “built environment”)]. The search scope was limited to journal articles indexed in CSSCI and CSCD. Both searches covered the period from 2015 to 2025.

### 2.3 Bibliometric analysis

This paper employs bibliometric analysis and uses CiteSpace 6.2 software for bibliographic visualisation. Through three complementary analytical methods, it explores the knowledge structure and evolutionary trajectory of research on community renewal oriented toward active ageing. First, keyword co-occurrence analysis is used to identify major research hotspots and the knowledge structure within this field. Node size represents keyword occurrence frequency, whereas link strength represents co-occurrence relationships between keywords. Second, a timeline analysis was conducted to examine the spatiotemporal evolution of research themes and emerging research frontiers. The position of each keyword marks the year of its first appearance in the literature, thereby illustrating the temporal evolution in research focus.

Finally, keyword clustering analysis was employed to identify thematic clusters and their interrelationships. Cluster quality was assessed using modularity (*Q*) and the silhouette coefficient (*S*), both of which are widely used metrics for evaluating network structure and clustering reliability. In this study, the analysis yielded a modularity *Q* of 0.76 and an average silhouette S of 0.89, both exceeding the conventional reliability thresholds (*Q* > 0.3, *S* > 0.7), indicating a well-defined and statistically reliable clustering structure (see also Section 4).

The time span for all analyses was set from 2015 to 2025, with one-year intervals. Keywords were selected as node types, and the top 50 keywords were retained for each time slice. The Pathfinder and minimum spanning tree algorithms were applied to simplify the network structure and enhance visualisation clarity. In addition, citation burst detection was performed using Kleinberg’s algorithm as implemented in CiteSpace 6.2 (*γ* = 0.5, minimum burst duration = 1 year). A keyword was classified as exhibiting a significant burst only when it met CiteSpace’s default significance threshold. The resulting burst onset years were used as the primary quantitative criterion for periodisation, as reported in Section 3.2 and Table 1. Before bibliometric analysis, keyword normalisation was conducted to improve semantic consistency. Spelling variants (e.g., “active ageing” and “active aging”), synonymous expressions (e.g., “older adults,” “elderly” and “older people”), abbreviations and semantically equivalent keywords were manually standardised according to a predefined keyword normalization protocol. The normalised dataset was subsequently imported into CiteSpace for keyword co-occurrence, clustering, timeline and burst analyses.

**Table 1:** Quantitative clustering of citation burst onset years from CNKI and WoS datasets (2015–2025).

Development phase and time window	Dataset source	Burst keyword (Original/English)	Burst strength	Begin–End	Domain paradigm & strategic focus
Phase I: Facility Expansion & Physical Intervention (2015–2018)	WoS	EIP (Active and Healthy Ageing)	2.35	2015–2017	Physical Space & Baseline Infrastructure Focuses on physical hardware retrofitting, spatial quality improvements, and technical adaptation standards (e.g., accessibility, public facilities)
		Active and healthy ageing	2.08	2015–2018	
		Gentrification	3.48	2016–2018	
		Regeneration	2.79	2017–2019	
		Land use	2.42	2018–2020	
	CNKI	创意社区 (Creative Community)	3.77	2015–2016	
		改造方法 (Renovation Method)	2.72	2015–2017	
		创意城市 (Creative City)	2.72	2015–2017	
		传统社区 (Traditional Community)	4.75	2016–2017	
		风景园林 (Landscape Architecture)	4.01	2018–2019	
		微更新 (Micro-regeneration)	3.26	2018–2020	
		剬共设施 (Public Facilities)	1.59	2018–2021	
Phase II: Behavioral Expansion & Social Governance (2019–2021)	WoS	Management	1.93	2019–2020	Social Pathways & Operational Governance Transitions from physical space to community governance mechanisms, emphasizing public participation, co-governance, and behavioral mediation
		Healthy aging	2.7	2020–2022	
		Active aging	2.66	2020–2022	
		Governance	2.66	2020–2022	
		Aging in place	1.67	2020–2021	
		Participation	2.76	2021–2022	
		Community	3.63	2021–2022	
	CNKI	多元共治 (Polycentric Governance)	1.9	2019–2020	
		剬众参与 (Public Participation)	0.41	2019–2021	
		微改造 (Micro-renovation)	3.92	2020–2021	
		党建引领 (Party-building Guidance)	0.97	2021–2023	
		加装电梯 (Elevator Retrofitting)	0.81	2021–2022	
Phase III: Multi-System Integration & Sustainability (2022–2025)	WoS	Climate change	1.63	2022–2023	Multidimensional Synergy & Ecological Systems Integrates climate resilience/low-carbon updates, smart digital technology, intergenerational inclusion, and empirical health performance evaluation
		Urban design	1.63	2022–2023	
		Redevelopment	2.38	2022–2023	
		Performance	3	2023–2025	
		People	1.49	2023–2025	
	CNKI	适老化 (Age-Friendliness)	1.18	2022–2023	
		低碳更新 (Low-Carbon Renewal)	0.39	2022–2023	
		乡村振兴 (Rural Revitalization)	0.75	2022–2023	
		智慧社区 (Smart Community)	2.12	2023–2025	
		农村老人 (Rural Elderly)	1.23	2023–2025	
		儿童参与 (Child Participation)	0.82	2023–2025	

All listed keywords satisfied CiteSpace’s default burst-significance threshold (*γ* = 0.5, Kleinberg’s algorithm); reported burst-strength values reflect the magnitude of each keyword’s frequency increase relative to its own citation baseline and are therefore not directly comparable in absolute terms across keywords with different overall frequencies.

After database retrieval, duplicate records were removed, and the remaining records were screened based on titles and abstracts. Subsequently, full-text eligibility assessment was conducted according to the predefined inclusion and exclusion criteria. The final dataset included 1,386 publications for bibliometric analysis. The detailed screening process and the number of records excluded at each stage are presented in Figure 1.

## 3 Results

### 3.1 Research hotspots

**Figure 2:**
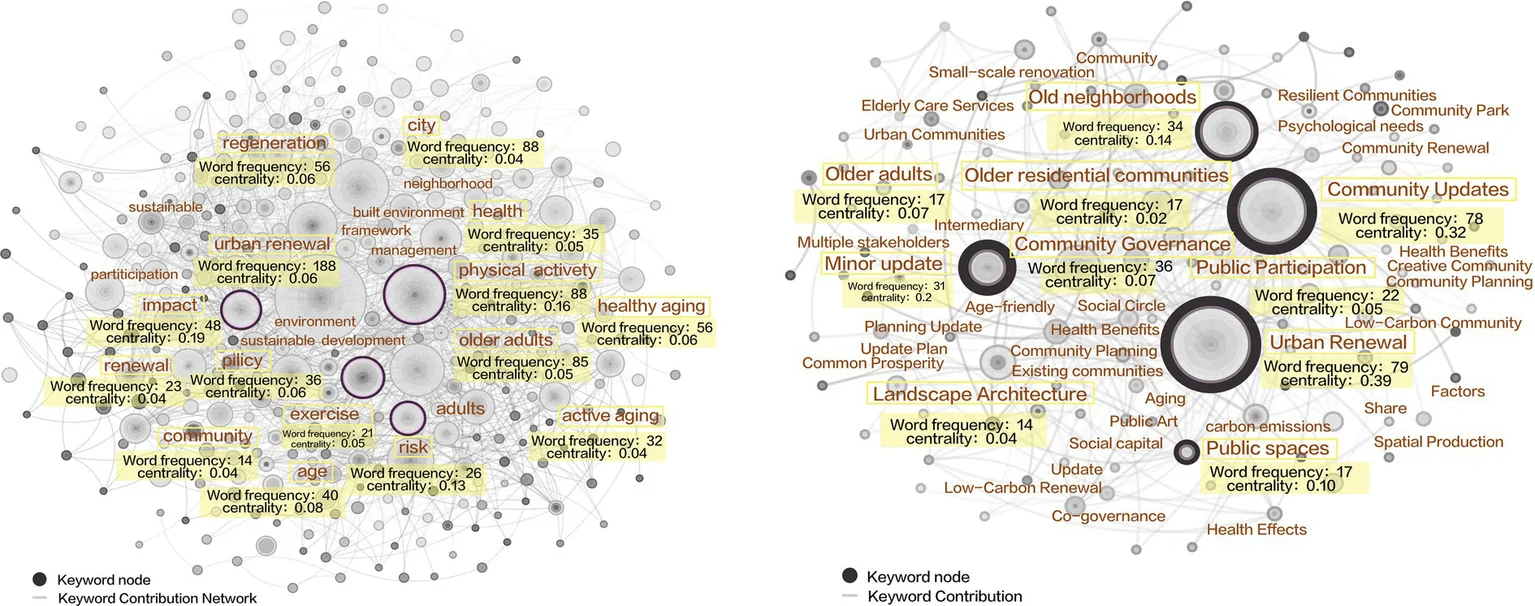
Keyword co-occurrence network of the international and Chinese literature.

Through co-occurrence analysis of keywords, core knowledge domains within this field were identified (Figure 2). The results reveal significant differences in research focus between international and Chinese studies. International research is primarily characterised by keywords such as “physical activity,” “health impacts,” and “risks,” indicating a strong emphasis on empirical validation of public health outcomes and environmental effects (3–5). The scope of research has gradually expanded from examining the impact of the built environment on physical activity to broader discussions on healthy ageing and health equity (16). In contrast, Chinese research is dominated by keywords such as “urban renewal,” “community renewal,” and “ageing communities.” These themes reflect a strong response to China’s national urban renewal policies and practical governance needs (12, 17). Compared to the international literature, Chinese research places greater emphasis on implementation of renovations, governance mechanisms, and policy application.

Despite these differences, both types of literature demonstrate a growing trend toward interdisciplinary integration (17). Although the research priorities differ, both knowledge systems increasingly converge on the need to integrate environmental, behavioural and governance perspectives to address the complex challenges of active ageing.

### 3.2 Thematic evolution

**Figure 3:**
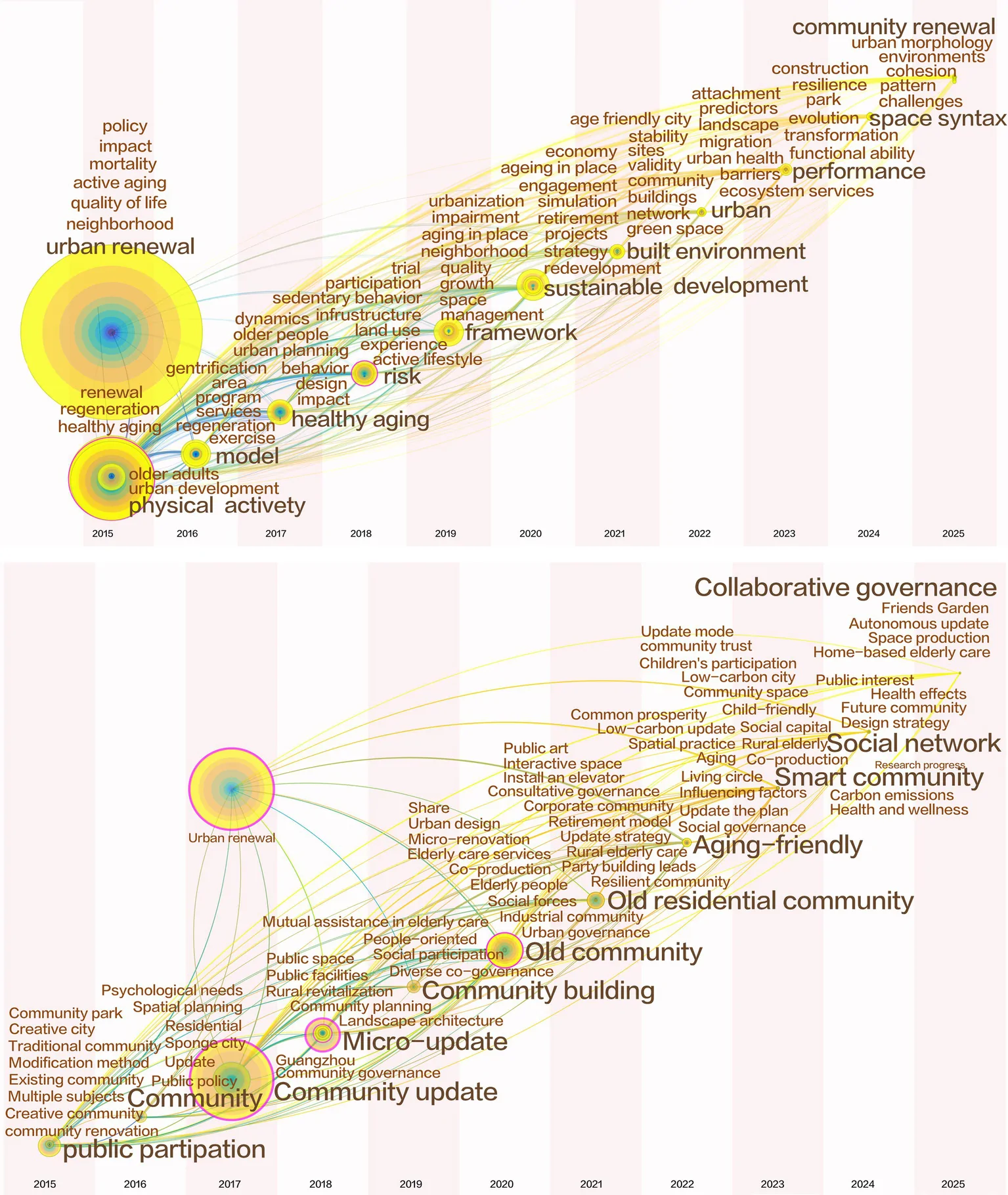
Keyword time-zone visualisation of the international and Chinese literature.

To eliminate potential periodisation bias, the three developmental phases were established based on the temporal clustering of keyword citation burst onset years using Kleinberg’s algorithm in CiteSpace (Table 1 and Figure 3). The empirical data across both CNKI and WoS datasets reveal three distinct transition windows based on burst onset patterns: Phase I (2015–2018, Facility Expansion Phase) is characterised by bursts concentrated on physical environment modifications and infrastructure standards, such as Renovation Method (2015), Land Use (2018) and Public Facilities (2018); Phase II (2019–2021, Behavioural Expansion Phase) marks a synchronised shift towards social pathways and operational governance across both databases, evidenced by keywords such as Polycentric Governance (2019), Management (2019), Governance (2020) and Participation (2021); and Phase III (2022–2025, System Integration Phase) is interpreted as reflecting multi-dimensional integration incorporating ecological sustainability (Climate Change, 2022; Low-carbon Renewal, 2022), smart technologies (Smart Community, 2023) (18) and quantitative health evaluation (Performance, burst strength = 3.00). This cross-database alignment of burst onset years indicates that the periodisation may be understood as an evidence-informed analytical interpretation of domain evolution, rather than a definitive classification of disciplinary development.

By incorporating a temporal dimension into the keyword co-occurrence network and using the “Timezone View” feature to generate a keyword co-occurrence timeline (Figure 3)—where the time associated with each node represents the year the keyword first appeared—and by analysing the coupling relationships between domestic and international policy nodes, three distinct phases of evolution can be identified. Based on the temporal distribution of keyword bursts, policy milestones and thematic evolution identified through bibliometric analysis, the three-phase framework proposed in this study should be understood as an evidence-informed analytical interpretation of research development rather than a definitive classification of disciplinary evolution.

Facility Expansion Phase (2015–2018): International research incorporated environmental psychology and evidence-based medicine methodologies, focusing on validating the correlation between the built environment and health indicators for older adults (19); Chinese research, meanwhile, concentrated on technical standards and engineering approaches for ageing-in-place retrofits in older residential communities (20). The core objective was to address the basic needs arising from physiological decline in older adults through spatial interventions, with barrier-free construction and elevator retrofitting representing the primary instruments of this phase. This phase treated older adults’ behaviour as a passive outcome of the built environment, neglecting the sociopsychological factors underlying behavioural generation and failing to explain variations in usage efficiency under similar renovation conditions (21).

Behavioural Expansion Phase (2019–2021): International research integrated built environment assessments with health impact measurement methods, beginning to identify the scope of effects where environment, behaviour, and health serve as mediating variables (22); Chinese research synchronized governance effectiveness evaluations and health performance assessments within ageing-friendly renovation practices (23), with studies now generally covering analyses across both spatial form and social governance dimensions. The core shift moved from the adequacy of spatial interventions to enhancing spatial usage efficiency. The limitations of single-space retrofitting gradually became apparent, driving research toward explaining Healthy Ageing through mediating variables. Barnett et al. (3) and van Cauwenberg et al. (24) confirmed through large-scale empirical studies that the built environment does not directly generate health benefits; rather, these benefits are realized through behavioural pathways that encourage sustained physical activity and promote neighbourhood interaction. This phase introduced social capital theory to explain variations in spatial utilization efficiency; however, it still failed to account for disparities in health outcomes under similar spatial and social conditions.

System Integration Phase (2022–2025): Domestic and international research, centered on the goal of active ageing, began integrating knowledge systems related to spatial planning, social governance, and ecological regulation to explore pathways for multidimensional synergy (25). Empirical studies have found that even when spatial optimization and social interventions are advanced simultaneously, significant disparities in health outcomes among older adults across different communities persist, prompting a shift toward multi-factor system integration (26). This phase begins to incorporate ecological and environmental factors, exploring pathways for synergy among the built environment, public services, governance systems, and green spaces (27), gradually forming a multi-dimensional research framework that intersects spatial, social and ecological dimensions (28).

Across both datasets, all identified burst keywords (100%) have onset years falling within their assigned phase window (17 from WoS and 18 from CNKI; see Table 1). More importantly, the two datasets—compiled independently and reflecting distinct international and domestic research traditions—each display the same tripartite clustering pattern (WoS: 5/7/5; CNKI: 7/5/6 keywords across Phases I/II/III respectively), providing convergent, cross-database quantitative support for the proposed periodisation rather than relying on visual interpretation of the timezone view alone.

### 3.3 The evolutionary logic of interdisciplinary development

By synthesising the results of keyword co-occurrence analysis and temporal analysis, the evolutionary trajectory of interdisciplinary development becomes evident. This field has gradually evolved from facility-oriented spatial interventions towards spatial–social behavioural transformations, and ultimately to the integration of spatial, social and ecological dimensions. The observed evolutionary trajectory suggests a gradual shift in research emphasis from facility-oriented interventions towards integrated spatial, social and ecological perspectives. This interpretation is derived from bibliometric evidence and policy context rather than as the only possible classification of disciplinary development.

This evolutionary process is interpreted as reflecting a fundamental shift in research understanding. Early studies primarily focused on providing physical facilities and improving the environment (20). Subsequent research recognised that social interactions and governance mechanisms are crucial for translating environmental conditions into behavioural change (22, 23). Recent studies further highlight that ecological and restorative environmental qualities play a key role in shaping sustainable health outcomes (11, 27). This evolutionary trajectory indicates that community renewal oriented toward active ageing has gradually shifted from a single-discipline perspective toward an integration of health effects. However, the spatial, social and ecological dimensions are viewed as interrelated but have rarely been theorised as sequentially connected mechanisms. This also lays the foundation for the theoretical discussion and framework development described below.

The bibliometric comparison between the international and Chinese literature reveals a clear asymmetry in research priorities. International studies are primarily organized around health mechanisms and behavioural outcomes, with high-frequency keywords such as physical activity, health impacts, risk, and built environment, indicating a mature theoretical emphasis on hypothesized pathways linking environmental interventions to health promotion. By contrast, Chinese studies are more strongly oriented toward policy implementation and urban governance, with keywords including urban renewal, community renewal, ageing communities, and old residential neighbourhoods, reflecting the influence of recent national urban regeneration strategies.

Although both streams contribute valuable knowledge, neither adequately integrates theory and practice within the governance context of high-density older residential communities in China. International evidence provides transferable theoretical mechanisms but pays limited attention to China’s institutional and spatial conditions, whereas Chinese studies remain closely aligned with policy agendas but lack an integrated theoretical framework capable of explaining how spatial interventions may contribute to health outcomes. This asymmetry, identified directly through our bibliometric analysis, provides an evidence-informed rationale for developing the Spatial–Social–Ecological (SSE) framework proposed in the following section, which is specifically intended to bridge theoretical advances with the practical demands of community renewal in China.

It should be clarified that ‘high-density’ does not emerge as a discrete keyword cluster within the bibliometric results; rather, it is interpreted as reflecting a well-documented structural characteristic of China’s pre-2000s urban residential stock, where plot ratios and building coverage are substantially higher than in most international comparison contexts (29). The bibliometric asymmetry identified above—Chinese research’s orientation toward ‘old residential neighbourhoods’ and governance implementation—thus is considered to be most applicable to this high-density housing form, which constitutes the dominant typology of ageing communities in urban China.

## 4 Discussion: thematic analysis and theoretical integration deficiency

Building upon the bibliometric findings presented in Section 3, particularly the identified asymmetry between internationally theory-oriented research and domestically policy-oriented research, this study synthesizes existing theoretical perspectives into an evidence-informed Spatial–Social–Ecological (SSE) conceptual framework for high-density older residential communities in China. Rather than proposing a new theoretical model, the framework integrates internationally established theories with China’s community renewal practices to provide a conceptual basis for interpreting the relationships identified through the bibliometric analysis. The proposed framework should therefore be understood as an evidence-informed conceptual synthesis that requires future empirical validation rather than as an empirically verified causal model. The necessity of the SSE framework derives from the specific characteristics of Chinese high-density older residential communities. Unlike communities where spatial resources and governance systems may already support ageing-friendly environments, these communities often require simultaneous improvements in physical accessibility, social coordination, and ecological quality under conditions of limited land resources and constrained renewal capacity.

Based on the CiteSpace clustering results (modularity *Q* = 0.76 > 0.3; average silhouette *S* = 0.89 > 0.7), which indicate a statistically reliable clustering structure, and by comparatively interpreting the clustering characteristics of the international and Chinese literature (Figure 3), three major thematic orientations may be identified across the existing research on community renewal for active ageing. International clusters “#5” and “#6” and Chinese clusters “#4” and “#8” primarily focus on the relationship between facility supplementation and older adults population; international cluster “#0” and Chinese clusters “#0,” “#2,” and “#3” concentrate on community governance and behavioural transformation; International clusters “#2” and “#4” and Chinese cluster “#1” revolve around the coordinated development of sustainable health systems, respectively corresponding to the three major mainstream themes in interdisciplinary research on active ageing and community renewal: verification of Healthy Ageing under facility supplementation; the role of behavioural transformation in community governance pathways; and the coordinated construction of sustainable Healthy Ageing.

### 4.1 Verification of the health effects of improved facilities

The improvement of facilities is the earliest and most well-established research area in the field of community renewal aimed at active ageing. Guided by the World Health Organization’s (WHO) Healthy ageing Agenda, research in this phase primarily focuses on whether improvements to age-friendly environments can yield quantifiable health benefits for older adults (1).

Existing research indicates that barrier-free infrastructure, walkable neighbourhood networks, accessible public services, and age-friendly public spaces are all positively correlated with older adults’ physical activity, mobility, and quality of life (3, 5, 30). By reducing environmental barriers and improving accessibility, spatial interventions create more opportunities for older adults to participate in outdoor activities and community life (24). Consequently, the provision of such facilities is widely regarded as the environmental foundation of active ageing (31).

However, although these associations have received substantial empirical support, facility-based research remains limited in its explanatory power. First, most such studies assume that health outcomes stem directly from environmental improvements, without sufficiently addressing the behavioural processes through which environmental opportunities translate into observed health benefits (21). Second, these studies often treat older adults as a homogeneous group, overlooking differences in functional abilities, lifestyle preferences, and social resources. Finally, studies have repeatedly shown that significant variations in health outcomes exist even within communities with similar standards of physical improvements, suggesting that spatial conditions alone cannot fully explain the processes underlying health outcomes.

These limitations indicate that physical interventions alone are insufficient to fully understand the relationship between community renewal and healthy ageing. Consequently, the question is no longer whether facilities are important, but rather how environmental improvements translate into sustained behaviours and health outcomes.

### 4.2 Community governance and behavioural transformation mechanisms

In response to the interpretive limitations of facility-oriented research, subsequent studies have increasingly shifted their focus to social and behavioural dimensions. The core research focus lies in understanding how the built environment translates into behavioural practices that yield health benefits. Existing literature indicates that spatial interventions create opportunities for action, but these opportunities must be activated through governance arrangements, social participation, and community interaction (7, 22). At the governance level, collaborative institutional arrangements can improve service accessibility and lower barriers to participation (32). At the social level, neighbourhood interaction, place attachment, and collective activities can enhance older adults’ willingness to use community resources (8, 33). At the behavioural level, these mechanisms help translate environmental accessibility into regular physical activity, social engagement, and community participation (22).

This shift may be understood as representing a significant theoretical advancement, as it recognizes that health outcomes are not directly generated by physical space but manifest through behaviour processes mediated by social factors. Consequently, health benefits depend not only on the facilities provided but also on how residents interact with and use them. Nevertheless, the spatio-social framework remains incomplete. While it successfully helps explain the phenomenon of behavioural activation, it struggles to account for why significant disparities in health outcomes persist across communities with similar levels of governance capacity and participation. This suggests that mechanisms beyond the spatial and social embedding are at play. Consequently, research is increasingly turning to ecological and restorative perspectives to seek a more comprehensive explanation for the generation of health effects.

### 4.3 Ecological healing and sustainable health outcomes

The growing recognition of ecological influences may be understood as representing the most recent development in active ageing-oriented community renewal research. Unlike earlier studies that focused on environmental provision or behavioural activation, this perspective examines how ecological qualities shape the sustainability and intensity of health outcomes.

Therapeutic Landscape Theory provides an important foundation for understanding this process (13). According to this perspective, health emerges through the interaction of physical settings, social experiences, and symbolic meanings. Complementary theories further explain the mechanisms involved. Attention Restoration Theory suggests that restorative environments replenish depleted cognitive resources (14), while Stress Reduction Theory indicates how exposure to natural environments promotes physiological recovery and emotional wellbeing (34).

Building upon these theoretical foundations, recent studies increasingly emphasize the role of green infrastructure, restorative environments, environmental comfort, biodiversity, and ecosystem services in promoting healthy ageing (20, 27). Ecological qualities not only encourage outdoor activity and social interaction but also directly influence psychological restoration and subjective wellbeing (35). Consequently, health outcomes are increasingly understood as the result of interactions among spatial conditions, social processes, and ecological experiences.

More importantly, ecological perspectives fundamentally alter the analytical focus of the field. Rather than treating health as an immediate outcome of environmental intervention, they conceptualize health generation as a long-term and dynamic process shaped by multiple interacting systems (36). This transition marks the emergence of a more holistic understanding of active ageing-oriented community renewal.

### 4.4 From multidimensional integration to the deficiency of theoretical integration

The preceding discussion reveals a clear pattern of theoretical evolution. Research has progressively expanded from spatial intervention, to social mediation, and finally to ecological health promotion. Each stage emerged in response to the explanatory limitations of the previous one, producing an increasingly comprehensive understanding of health generation within community renewal contexts.

Despite this progress, a critical theoretical problem remains unresolved. Existing studies generally acknowledge the importance of spatial, social and ecological factors, yet these dimensions are typically treated as parallel determinants rather than sequentially connected mechanisms (37). As a result, the field lacks a coherent explanation of how spatial adaptation creates behavioural opportunities, how social processes transform those opportunities into active engagement, and how ecological qualities sustain or amplify resulting health outcomes.

This limitation is interpreted as reflecting the absence of a theoretically integrated explanatory architecture capable of linking spatial adaptation, social embedding, ecological healing, and health outcomes. Consequently, although multidimensional integration has become a dominant research trend, the mechanisms connecting these dimensions remain insufficiently specified and operationalized.

**Figure 4:**
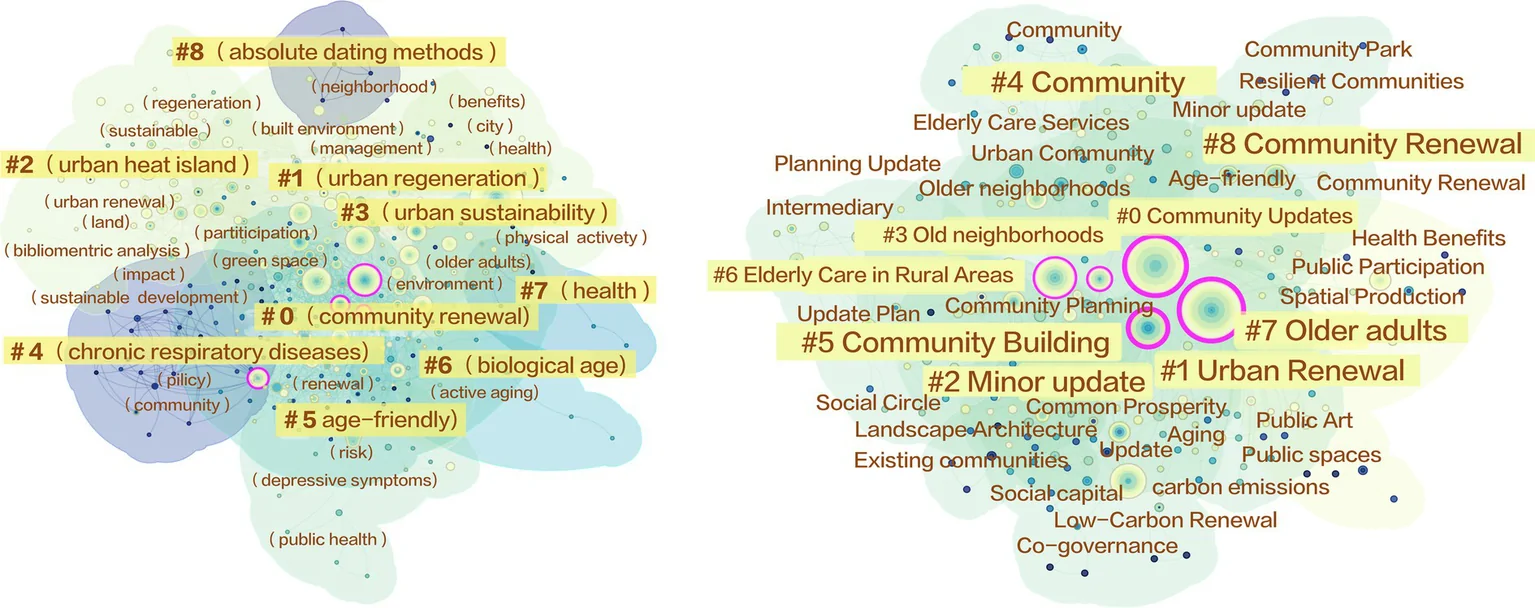
Keyword clustering map of the international and Chinese literature.

This lack of integration is not merely a methodological limitation; it is interpreted as reflecting a deeper theoretical gap—the absence of a theoretical framework capable of elucidating the connections between the Spatial Adaptation (enabling conditions), the Social Embedding (mediating mechanisms), and the ecological dimension (regulatory outcomes). The SSE framework proposed in Section 4.5 is intended to provide a conceptually integrated interpretation of this theoretical gap. Its three complementary dimensions are informed by the three major stages identified through the bibliometric analysis rather than representing empirically verified sequential mechanisms: its three conceptual dimensions are informed by the three major developmental phases identified through the bibliometric analysis—the Facilities phase reveals the necessity of spatial prerequisites (the Spatial Adaptation dimension); the behavioural stage reveals the necessity of social mediation (the Social Embedding dimension); and the ecological stage reveals the necessity of restorative regulation (the Ecological Healing dimension). Thus, this framework may be understood as representing a theory-based synthesis of accumulated practice in the field, rather than a simple superimposition of existing models (see Figure 4).

### 4.5 Synthesising existing theoretical perspectives into an evidence-informed Spatial–Social–Ecological conceptual framework

**Figure 5:**
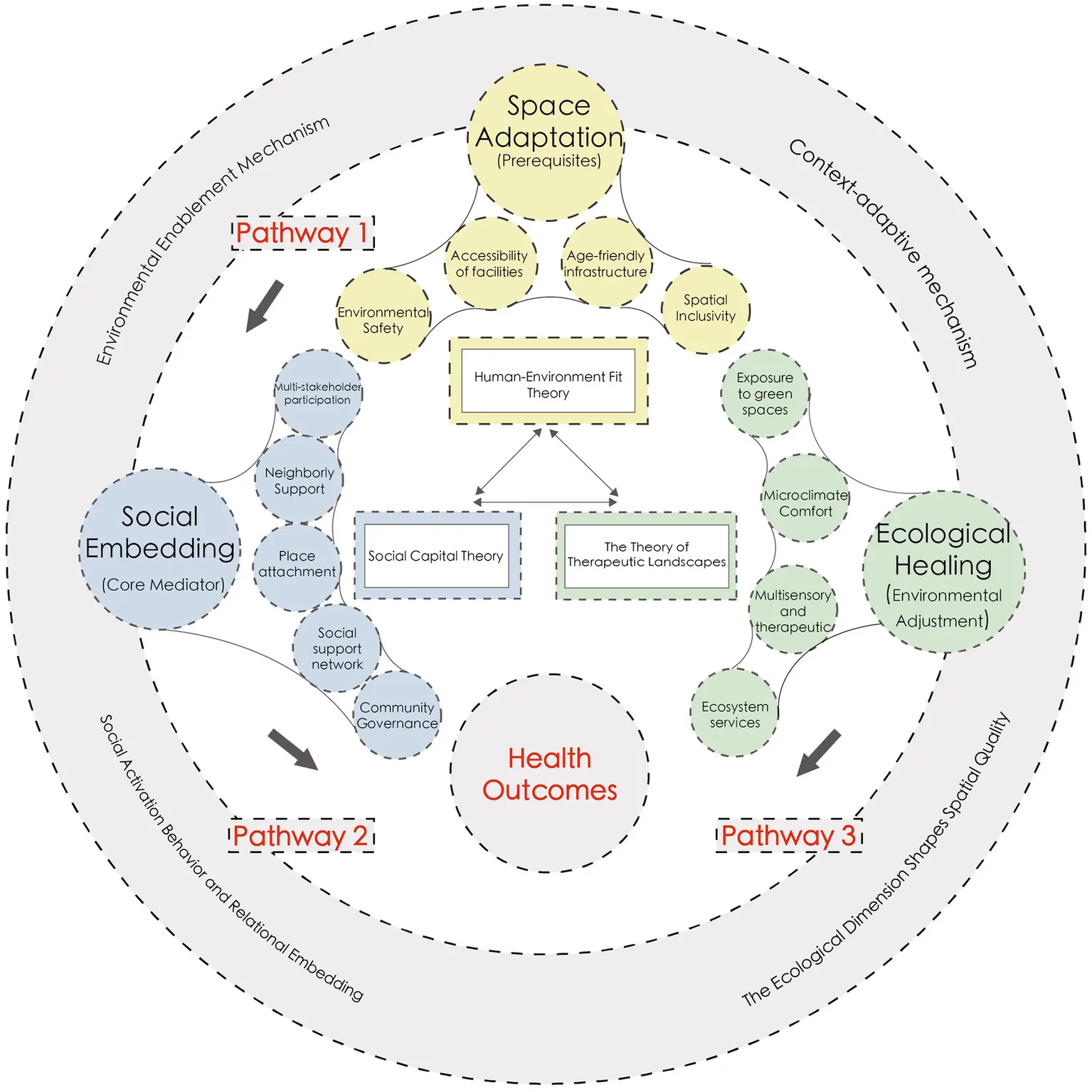
Proposed Spatial–Social–Ecological (SSE) conceptual framework illustrating hypothesised theoretical relationships among spatial adaptation, social embedding, ecological healing and healthy ageing outcomes. Solid arrows (P1, P2) indicate hypothesised sequential pathways; the dashed arrow (P3) indicates a hypothesised direct pathway. Ecological Healing is conceptualised as a complementary dimension that may contribute to health outcomes through restorative environmental qualities, alongside the behavioural pathway.

The Spatial–Social–Ecological (SSE) framework proposed in this study is intended as an evidence-informed conceptual framework synthesized from bibliometric findings and established theoretical perspectives. The relationships illustrated in Figure 5 represent hypothesized theoretical pathways derived from bibliometric synthesis and established theoretical perspectives rather than empirically verified causal mechanisms. Accordingly, these proposed relationships should be regarded as a foundation for future hypothesis testing using longitudinal, experimental, or structural equation modelling approaches. Within the proposed SSE framework, spatial adaptation, social embedding, and ecological healing are conceptualized as three complementary theoretical dimensions. Rather than representing statistically verified mediating or moderating variables, each dimension contributes conceptually to explaining how community environments may support healthy ageing through different but interrelated processes.

Building on the bibliometric analysis and thematic discussions presented in Sections 3 and 4, this section shifts the research focus from a descriptive literature bibliometric review to the development of an explanatory framework, with a particular emphasis on exploring how environmental improvements translate into health outcomes for older adults through identifiable theoretical pathways. By integrating spatial, social and ecological elements into a unified analytical model, the SSE framework proposes a set of interconnected theoretical pathways linking environmental conditions, behavioural transformation, restorative processes, and health outcomes (Figure 5). Figure 5 illustrates the proposed theoretical relationships linking spatial adaptation, social embedding, ecological healing, and healthy ageing outcomes. Pathway 1 (Spatial Adaptation-Social Engagement): spatial prerequisites may enable behavioural opportunities available to older adults by reducing environmental barriers. Pathway 2 (Social Embedding-Health Outcomes): social mechanisms-governance structures, neighbourhood networks, and place attachment-may facilitate the translation of spatial opportunities into behavioural engagement potentially associated with health outcomes. Pathway 3 (Ecological Healing–Restorative Mechanisms): ecological characteristics may contribute to healthy ageing through restorative environmental qualities, including restorative perception and physiological stress regulation, while complementing spatial and social mechanisms.

#### 4.5.1 Theoretical foundations of the SSE framework

The bibliometric evidence and thematic synthesis presented in Sections 3 and 4 indicate that existing studies have progressively expanded from facility-oriented spatial interventions to socially mediated behavioural mechanisms and, more recently, to ecological health promotion.

Nevertheless, these three dimensions have generally been examined as parallel explanatory factors rather than as sequentially connected mechanisms. This theoretical discontinuity provides the rationale for developing the Spatial-Social-Ecological (SSE) framework. Rather than introducing additional variables, the framework reorganizes existing evidence into an evidence-informed conceptual structure in which spatial adaptation provides behavioural opportunities, social embedding facilitates behavioural participation, and ecological healing is hypothesized to contribute to the sustainability of healthy ageing.

Within the social embedding dimension, Social Capital Theory provides the principal theoretical basis for explaining how spatial opportunities are translated into sustained behavioural engagement. Following Putnam (36) and Woolcock (38), the framework distinguishes three complementary forms of social capital—bonding, bridging, and linking—each representing a different type of social relationship operating within community environments. Bonding capital refers to strong horizontal ties among residents sharing common identities and everyday interactions. Bridging capital is interpreted as reflecting connections across heterogeneous groups that facilitate broader social participation and collective action. Linking capital may be understood as representing vertical relationships between residents and formal institutions, including community organizations, neighbourhood committees, and local governments, through which residents gain access to public resources and governance support.

Based on this theoretical distinction, place attachment is classified as an indicator of bonding social capital rather than linking social capital. Place attachment describes residents’ emotional, cognitive, and behavioural bonds with their living environment and is interpreted as reflecting a shared sense of belonging developed through long-term everyday interactions within the community. According to Scannell and Gifford’s tripartite model (39), place attachment comprises person, place, and psychological process dimensions, emphasizing emotional identification with place rather than institutional relationships. Because it strengthens internal community cohesion instead of facilitating vertical access to institutional resources, place attachment is theoretically more consistent with bonding social capital.

Accordingly, the five indicators of the social embedding dimension are reorganized as follows. Bonding social capital is represented by neighbourhood mutual aid, social support networks, and place attachment; bridging social capital is reflected by participation in multi-stakeholder governance and broader community interaction; linking social capital is represented by the quality of community governance systems and institutional collaboration. This revised classification improves the conceptual consistency of the SSE framework by ensuring that each indicator corresponds directly to its original theoretical definition.

#### 4.5.2 Theoretical foundations of the spatial, social and ecological dimensions

(1) Person–Environment Fit Theory was selected over alternative environmental competence theories—including Bronfenbrenner’s Ecological Systems Theory and Lawton’s broader environmental docility hypothesis—because it specifically operationalizes the competence-press dynamic at the individual-environment interface, providing the most direct theoretical basis for specifying spatial prerequisites for behavioural agency among functionally heterogeneous older adult populations. The Person–Environment Fit Theory (37) defines the boundaries of behavioural agency by establishing the principle that health-related behaviours emerge only when environmental demands do not exceed an individual’s capabilities. Contemporary operationalisations of this theory have been validated by Wahl, Iwarsson and Oswald (40). This theory provides the theoretical basis for the types of spatial adaptation variables within the SSE framework and operationalizes them through four quantifiable indicators: environmental safety, accessibility to facilities, spatial inclusivity, and the provision of age-friendly infrastructure (41). These indicators constitute the exogenous variables of the framework—which can be directly addressed through age-friendly renovations—and serve as necessary preconditions for behavioural participation (42).

(2) Social Capital Theory was selected over Social Exchange Theory and Community Resilience frameworks because it specifically articulates the relational mechanisms—bonding capital, bridging capital, and institutional trust—through which social embeddedness converts spatial opportunity into realized behavioural engagement, without requiring the assumption of rational utility maximization. Subsequent developments by Woolcock further extended the analytical application of social capital to institutional and governance contexts (36). Contemporary applications to ageing and community health have been extensively developed by Nyqvist et al. (43) and Forsman et al. (42). Within the SSE framework, the social embedding dimension is operationalized through five measurable indicators that correspond to distinct forms of social capital. Bonding social capital is represented by neighbourhood mutual aid, social support networks, and place attachment, all of which strengthen residents’ sense of belonging and internal community cohesion. Bridging social capital is reflected in participation in multi-stakeholder governance, which promotes interaction across different social groups. Linking social capital is represented by the quality of community governance systems, reflecting residents’ relationships with formal institutions and their access to governance resources (42).

(3) Therapeutic Landscape Theory was selected over Biophilic Design Theory and Salutogenic Theory because it uniquely integrates physical, social, and symbolic environmental dimensions within a single analytical framework, directly paralleling the SSE structure, while ART and SRT provide complementary mechanistic explanations for its cognitive and physiological pathways, respectively. The Theory of Therapeutic Landscapes, together with Attention Restoration Theory (ART) and Stress Reduction Theory (SRT), provides the conceptual basis for understanding how restorative environmental qualities may complement the spatial and social dimensions of the SSE framework by contributing to healthy ageing through psychological restoration and physiological stress reduction.

Within the SSE framework, this theoretical cluster dominates the category of ecological healing variables and is operationalized through four measurable indicators: green space access, microclimate comfort, multisensory restorative qualities, and ecosystem services. These indicators represent the ecological dimension of the SSE framework and are intended to complement rather than replace the spatial and social dimensions when interpreting healthy ageing (44). Collectively, these three theoretical traditions form a theoretically integrated explanatory system rather than a simple disciplinary overlay. The Person–Environment Fit Theory proposes the spatial constraints that limit behavioural possibilities; Social Capital Theory identifies the social relational mechanisms that transform possibilities into observed participation; and the Therapeutic Landscape Theory, grounded in ART and SRT, clarifies the ecological conditions that may strengthen the effectiveness of behavioural engagement in promoting healthy ageing. The resulting SSE framework integrates these three complementary theoretical dimensions into a coherent conceptual structure in which each dimension is hypothesized to contribute distinct explanatory perspectives. To enhance the framework’s empirical testability, this operationalization provides a direct foundation for subsequent structural equation modeling (SEM) studies of the theoretical pathways proposed by the SSE framework, as well as hierarchical linear modeling (HLM) studies of cross-community variability in health outcomes. Ecological healing represents the ecological dimension of the SSE framework. It is proposed to influence healthy ageing through restorative environmental qualities, including green space accessibility, environmental comfort, multisensory experiences, and ecosystem services. Rather than functioning as a statistically defined moderator, Ecological Healing is conceptualized as a complementary theoretical component that operates alongside spatial adaptation and social embedding within the overall explanatory framework.

Three boundary conditions govern the SSE framework’s applicability. First, Person–Environment Fit Theory was originally developed in the context of institutionalized care environments; its extension to open community settings assumes that environmental demands in older residential communities are sufficiently varied to create meaningful competence-press differentials across residents. This assumption is supported by documented heterogeneity in older adults’ functional capacity across the 60–85+ age range but may be less applicable in communities where all residents exhibit similar functional profiles. Second, Therapeutic Landscape Theory’s extension from specialized healing sites to ordinary community spaces is justified by growing evidence that everyday natural and social environments generate restorative effects through the same ART and SRT mechanisms originally documented in dedicated healing contexts—provided that they possess sufficient restorative qualities. In low-quality ecological environments with minimal green space, this pathway may be attenuated or absent. Third, Social Capital Theory’s Western conceptualization of voluntary association-based bonding and bridging capital requires cultural adaptation in the Chinese context, where social capital formation occurs primarily through Party-led community governance structures and kinship networks rather than voluntary civic associations. The framework’s social embedding indicators have been adapted accordingly.

#### 4.5.3 Theoretical pathways and research hypotheses

Based on the SSE framework, three empirically testable hypotheses are proposed for future structural equation modelling validation. H1: Spatial adaptation provides the environmental prerequisites for healthy ageing by reducing environmental barriers and facilitating opportunities for social participation. H2: Social embedding functions as the principal mediating mechanism through which spatial opportunities are translated into sustained behavioural engagement and improved healthy ageing outcomes. H3: Ecological healing is hypothesised to contribute to healthy ageing through restorative environmental qualities, operating as a complementary pathway alongside spatial adaptation and social embedding.

#### 4.5.4 Implications for practice and policy

The integration of spatial, social and ecological dimensions within the SSE framework represents a theory-informed attempt to address the theoretical integration deficiencies identified in prior research. Ecological characteristics are hypothesized to complement spatial adaptation and social embedding by strengthening restorative environmental conditions associated with healthy ageing.

Compared with existing research that predominantly identifies variables or documents correlations, the SSE framework specifies proposed directionality, operationalizes 13 measurable indicators, and advances three testable hypotheses, providing both theoretical coherence and empirical tractability. For practitioners, the framework offers a prioritization logic for renovation investment: spatial accessibility improvements establish the necessary enabling conditions; governance mechanisms determine the extent to which those conditions are behaviourally activated; and Ecological Healing qualities may influence the extent to which behavioural engagement contributes to stable and sustainable health outcomes. The SSE framework thus provides an integrated tool for evidence-based ageing-friendly renovation policy in China’s high-density older residential communities. Rather than representing empirically established mechanisms, the framework proposes a set of theoretically informed relationships that require future empirical verification.

## 5 Conclusion

### 5.1 Key findings

Through a bibliometric analysis of 1,386 articles published between 2015 and 2025 on community renewal guided by active ageing, this study identifies three key findings.

First, the field exhibits distinct phased evolution. The bibliometric analysis reveals a coherent developmental trajectory spanning three phases: from one-dimensional spatial facility provision (2015–2018), to two-dimensional spatial-social behavioural transformation (2019–2021), and finally to three-dimensional spatial-social-ecological system integration (2022–2025). The observed transition between phases may be interpreted as reflecting an increasing tendency to address limitations identified in earlier research, suggesting a gradual shift from relatively low-dimensional perspectives towards more integrated theoretical interpretations.

Second, while Chinese and international research follow different trajectories, they are complementary. International academic research is primarily driven by theoretical validation and mechanistic explanations, advancing interdisciplinary analytical models through empirical testing. In contrast, Chinese research is policy-oriented, with research priorities adjusted in line with the national governance agenda—a pattern reflecting the dominance of practice-driven research over theoretical development in the Chinese context. This divergence highlights both the necessity of establishing a localized theoretical framework and the potential for evidence from Chinese policy practices to enrich theoretically developed models at the international level.

Finally, this study synthesizes an evidence-informed Spatial-Social-Ecological (SSE) conceptual framework that integrates the three complementary dimensions identified through the bibliometric analysis. The framework proposes three theoretically hypothesized pathways in which spatial adaptation provides behavioural opportunities, social embedding facilitates behavioural participation, and ecological healing is conceptualized as a complementary restorative dimension that may strengthen healthy ageing alongside spatial and social mechanisms. The proposed framework provides a conceptual foundation for future empirical validation rather than an empirically verified explanatory model.

### 5.2 Theoretical contributions

In terms of its relationship to existing theoretical traditions, this study makes three specific theoretical moves. First, it extends Therapeutic Landscape Theory beyond its original application to specialized healing sites (spas, pilgrimage sites, clinical settings) to ordinary urban community spaces—suggesting that the physical–social–symbolic interaction mechanisms identified by Gesler may also be applicable to everyday community environments of older residential communities when ecological quality is sufficient. This extension substantially broadens the theory’s empirical scope. Second, it re-contextualises Person–Environment Fit Theory from its original institutional care setting to open community environments, operationalising the competence-press dynamic through four renovation-specific spatial indicators that are directly amenable to policy intervention. Third, it culturally adapts Social Capital Theory to the Chinese governance context by respecifying bonding, bridging, and linking capital through Party-led multi-stakeholder governance structures, offering an empirically grounded modification of the theory’s Western voluntary-association assumptions for application in collectivist governance contexts.

### 5.3 Practical implications

In addition to its theoretical contributions, the SSE framework offers concrete guidance for the practice and policy of age-friendly retrofitting in China’s high-density, ageing residential communities. These implications are particularly relevant for Chinese high-density older residential communities, where limited spatial resources and complex governance structures require coordinated rather than single-dimensional interventions.

In terms of renovation planning, a step-by-step logic emerges: improvements in spatial accessibility form the foundational layer, thereby facilitating subsequent behavioural and ecological effects. Without adequate spatial prerequisites, the effectiveness of subsequent social and ecological interventions may be constrained. Therefore, renovation planning should first ensure basic spatial accessibility—barrier-free renovations, upgrades to walkable streetscapes, and age-friendly public space design—before allocating resources to higher-level social and ecological interventions.

Regarding community governance and management, the social embedding layer views Party-led multi-stakeholder collaboration, neighbourhood mutual aid networks, and the cultivation of local belonging as mechanisms for translating spatial possibilities into observed behavioural engagement (45). Community governance bodies should prioritize projects that activate these social mechanisms—particularly self-organized group activities, peer support systems, and opportunities for intergenerational interaction—as vital complements to physical renovations.

In terms of policy integration, the framework’s ecological healing dimension provides a potential analytical bridge between two parallel national policy strands that currently lack coordination: “China Urban Renewal Action Plan (2021–2025)” and “National Medium- and Long-Term Plan for Active Ageing (2021)” Future empirical studies may examine whether incorporating these indicators into community renewal evaluation systems can improve the assessment of health-related outcomes—thereby transforming renewal projects from spatial improvement initiatives into interventions with demonstrable health impacts. Within the present conceptual framework, Ecological Healing is primarily conceptualized as a complementary theoretical dimension that may enhance the effectiveness of spatial and social interventions, rather than as an independent determinant of health outcomes.

### 5.4 Limitations of the study

This study has three limitations. First, the analysis was limited to literature indexed in SSCI/SCIE/A&HCI and CSSCI/CSCD. While this ensured the quality of the research, it may have excluded relevant studies published in regional journals not included in these databases or written in languages other than English or Chinese. Although keyword normalization was performed to improve semantic consistency, bibliometric analysis remains subject to inherent limitations associated with database coverage, evolving terminology, and the continuous emergence of new research topics, which may influence knowledge mapping over time. Second, the SSE framework remains at the theoretical and conceptual level; the theoretical pathways and three empirical hypotheses it proposes have not yet been validated through empirical data collection. Rigorous structural equation modeling tests are required before definitive conclusions can be drawn. Finally, this framework was specifically constructed for high-density older residential communities in China; adjustments may be necessary when applying it to communities with different spatial configurations, governance structures, or demographic characteristics.

### 5.5 Directions for future research

For future research, this paper proposes four directions, all of which stem directly from unresolved issues within the SSE framework and the limitations identified above. Specifically, future empirical studies may examine the three theoretically proposed pathways individually before evaluating the overall SSE framework. First, empirical validation of the theoretical pathways within the SSE framework should be prioritized. Future empirical studies should incorporate both objective and subjective health indicators, including physical activity levels, mobility performance, psychological stress, loneliness, social participation, and quality of life. Conducting structural equation modeling using data from multiple communities at different stages of ageing-friendly retrofitting will provide the first rigorous test of the framework’s sequential theoretical architecture, Pathway 1 (Spatial Adaptation-Social Engagement): spatial prerequisites may enable behavioural opportunities available to older adults by reducing environmental barriers. Pathway 2 (Social Embedding-Health Outcomes): social mechanisms-governance structures, neighbourhood networks, and place attachment-may facilitate the translation of spatial opportunities into behavioural engagement potentially associated with health outcomes. Pathway 3 (Ecological Healing-Restorative Mechanisms): ecological characteristics are hypothesized to contribute to health outcomes by strengthening restorative experiences and enhancing the effectiveness of behavioural engagement.

Second, longitudinal and quasi-experimental study designs should be developed to provide stronger evidence for policy investment decisions. The current evidence base is almost entirely cross-sectional. In matched communities, comparing older adults’ health outcomes before and after age-friendly renovations through natural experiments would significantly advance the field’s understanding of causality and provide stronger evidence for policy investment decisions.

Third, the ecological healing dimension requires deeper empirical development. In the context of China’s high-density communities—which starkly contrast with the low-density, nature-rich environments in which Therapeutic Landscape Theory, ART, and SRT were originally developed—it remains empirically unclear which specific ecological features generate the strongest restorative effects. Research combining objective environmental measurements with physiological health monitoring would directly operationalize this dimension of the framework and generate context-specific design guidelines.

Fourth, comparative studies should examine the applicability of the SSE framework across different community types. Whether this framework can be extended to urban villages, work-unit communities, and suburban communities—each with unique spatial configurations, financial resources, and institutional arrangements—requires dedicated research. Such studies would not only test the framework’s universality but also generate the context-specific knowledge necessary for its implementation within China’s diverse urban landscape.

## Data Availability

The original contributions presented in the study are included in the article/supplementary material, further inquiries can be directed to the corresponding author.
